# Recurrent COVID-19 infection in a health care worker: a case report

**DOI:** 10.1186/s13256-021-02881-8

**Published:** 2021-07-12

**Authors:** Jaya Garg, Jyotsna Agarwal, Anupam Das, Manodeep Sen

**Affiliations:** Department of Microbiology, Dr. Ram Manohar Lohia Institute of Medical Sciences, Lucknow, 226010 India

**Keywords:** COVID-19, Health care worker, Recurrent, Reactivation, Reinfection

## Abstract

**Background:**

Recurrent coronavirus disease 2019 (COVID-19) infection is an emerging problem and may prove to be one of the greatest problems in controlling the pandemic in the future. Recurrent infections can be due to reactivation of dormant severe acute respiratory syndrome coronavirus 2 (SARS-CoV-2) or reinfection with similar or different strains of SARS-CoV-2.

**Case presentation:**

Here we present an interesting case of a health care worker working as a laboratory assistant at a COVID-19 laboratory who developed recurrent COVID-19 infection. He did not develop an immune response after the first episode of COVID-19; however, immunoglobulin G (IgG) antibodies were detected after the second episode.

**Conclusions:**

Through this case, we discuss the concept of reactivation and reinfection in the post-COVID period. We suggest that standard guidelines should be established to check for viral shedding and immune response among cured cases of COVID-19 after discharge via serial real-time polymerase chain reaction (RT-PCR) testing and IgG antibody detection. Further, strict hygiene practices should be stressed to these patients with possibility of COVID-19 recurrence.

## Background

The coronavirus disease 2019 (COVID-19) pandemic has resulted in substantial morbidity and mortality worldwide, along with widespread social and economic disruption. Every day new predictions and theories are evolving related to its virulence, clinical patterns, pathogenesis, prognosis, and treatment [1]. According to the World Health Organization (WHO), total 166 million cases of COVID-19 have been reported to date, with 3.5 million deaths. Of this total, India has contributed 26 million positive cases. Although the total number of positive cases is declining in certain countries such as England, France, Russia, and the Mediterranean countries, an alarming rise in the number of cases is occurring which may be due to new cases or recurrent infection in vulnerable populations [2]. A recent systemic review on re-positivity among 2436 recovered cases of COVID-19 reported that 16% of these patients were found to be positive again in follow-up, among which China and Korea accounted for 15% and 31%, respectively. Similar data have been documented worldwide, and re-positive cases are actually the tip of the hidden iceberg of this pandemic and need to be explored [3].

Recurrent or re-positive cases can be classified as reactivation with the same strain of severe acute respiratory syndrome coronavirus 2 (SARS-CoV-2) or reinfection with a different strain. We present the first case report of recurrent infection from India, discussing the case in the context of the existing debate surrounding reactivation versus reinfection.

## Case presentation

We present the case of a 30-year-old Indian male health care worker working as laboratory attendant at the COVID-19 lab at our institute. On 24 June 2020, he presented with fever and flu-like symptoms at the fever clinic. He was thoroughly examined by the physician, and in view of the ongoing COVID-19 pandemic, a real-time polymerase chain reaction (RT-PCR) test for COVID-19 was suggested. His nasopharyngeal and oropharyngeal swabs were collected in viral transport media (VTM) and immediately transported to the COVID-19 laboratory. RNA was extracted from the sample using the QIAamp Viral (Ribonucleic acid) RNA Mini Kit (Qiagen, Germany), and RT-PCR was performed using the LabGun COVID-19 RT-PCR Kit (Lab Genomics, Republic of Korea).

The RT-PCR results showed a cutoff threshold (ct value) for the envelope gene (E-gene) and RNA-dependent RNA polymerase (RdRP) gene of 21 and 20, respectively, indicating COVID-19 positivity. In view of the ongoing admission policy issued by the Ministry of Health and Family Welfare, New Delhi, India, in June 2020, he was immediately transferred to the COVID-19 ward of our hospital.

He was stable upon admission to the COVID ward. There was no significant medical history or history of any medical intervention. General physical examination showed no abnormalities; he was responding well to all commands and was cooperating with the treating physicians. No sign of breathlessness was seen, and oxygen saturation of 99% was maintained on room air. His chest X-ray, electrocardiogram (ECG), and routine investigations including complete blood count, liver function test, renal function test, and inflammatory markers including C-reactive protein, ferritin, lactate dehydrogenase, and coagulation profile were all within normal limits.

He was provided standard supportive treatment with paracetamol, vitamin C with zinc supplementation, and loading dose of hydroxychloroquine 400 mg twice a day followed by 200 mg once daily for 10 days. Repeat swabs were collected for real-time polymerase chain reaction (RT-PCR) on the 10th day and reported negative by the laboratory. A repeat sample was collected the next day and again returned negative results. In view of two negative RT-PCR results, he was discharged and advised to adhere to home quarantine for 1 week, and rejoined the duty after completing home quarantine. On day 30 after the initial diagnosis of COVID-19, a blood sample was collected for SARS-CoV-2 IgG antibody assay using the Abbott ARCHITECT chemiluminescent immunoassay (CLIA), and showed IgG antibody negativity. For the next 2 months the patient was healthy and experienced no post-COVID complications. However, approximately 90 days after his initial illness, he again developed flu-like symptoms with high-grade fever, severe myalgia, anosmia, and loss of taste. COVID-19 RT-PCR was again performed using the same kit, and showed positive results, with E-gene and RdRP cutoff threshold of 21 and 23, respectively.

General physical examination showed no abnormalities, and oxygen saturation of 100% was maintained on room air. His chest X-ray, ECG, and routine investigations were within normal limits. The patient expressed his unwillingness for hospital admission, and in view of the current Indian governmental policy he was advised to undergo home quarantine. He was provided supportive treatment and ivermectin 12 mg tablets taken orally once daily for 3 days. He responded well to the treatment, and on the 10th day of his illness, repeat samples for COVID-19 RT-PCR were negative. Currently, 6 months after reinfection, he is doing well; as a health care worker, he is actively involved in the COVID-19 laboratory. On day 30 of the second episode of COVID-19, a blood sample was retested for SARS-CoV-2 IgG antibody using the Abbott ARCHITECT CLIA, and this time the antibodies were detectable at a concentration of 2.6 at index (sample/calibrator), and were considered positive according to the manufacturer's recommendations (positive ≥ 1.4).

## Discussion and conclusions

The immune response to SARS-CoV-2 involves both cell-mediated and humoral immunity, but their long-term protective role is currently under evaluation. It is unclear whether and for how long infection with SARS-CoV-2 in humans protects from reinfection [1]. Research on the Middle East respiratory syndrome coronavirus and severe acute respiratory syndrome coronavirus has shown that neutralizing antibodies are induced and immunity wanes at around 18 months and an average of 2 years, respectively, suggesting that COVID-19 infection will confer at least "short-term" immunity, so that one would be unlikely to be infected again in the current season [4]. Furthermore, we now know from nonhuman primate models that infection with SARS-CoV-2 does protect from reinfection for at least some time [5]. We also know that the transfer of the serum of convalescent animals or neutralizing monoclonal antibodies to naïve animals can be protective and reduces virus replication significantly. However, experts note that antibodies may not be produced by every individual, or, if they do develop, they may not last long enough, therefore allowing the virus to enter the body and cause the disease again, assuming that antibodies triggered by COVID-19 are short-lived, leaving patients susceptible to recurrent infection.

Here we present an interesting case of a health care worker from India who had a mild case of COVID-19 but did not develop antibodies against SARS-CoV-2. A few months later he had a second episode of COVID-19 and developed antibodies against SARS-CoV-2. This second episode could have been reactivation or reinfection, and the role of antibodies in protection against recurrent COVID-19 infection is still controversial. In India, there are no documented cases of reinfection or reactivation in the medical literature, but there are a few unverified cases reported in Indian newspapers [6–8], and the interval reported between episodes of two infections is 30 to 40 days. Similar cases have been reported in the international literature. For example, recurrent infection was reported in Brazil, where the interval between the onset of symptoms in the two episodes ranged from 53 to 70 days (median, 56.5 days) [9]. Yuan *et al.* showed that 14.5% (25/172) of discharged COVID–19 Chinese patients had recurrent infection. This proportion has been reported between 9 and 21% in other studies [10]. A recent systematic review and meta-analysis including 38 studies on recurrent COVID-19 infection reported 466 recurrent cases worldwide, including cases from China (*n*=346), Korea (*n*=83), Iran (*n*=1), Italy (*n*=3), France (*n*=11), Brazil (*n*=1), and the United States (*n*=1) [3].

Recurrent COVID 19 infection is a new clinical entity and is rarely diagnosed. It can occur due to reactivation of primary infection or reinfection by SARS-CoV-2 in patients who fail to develop antibodies against primary infection. Reactivation is due to dormant virus which persists in the body and is activated. The virus can become dormant and later reactivate itself. While our immune system is able to clear most pathogens, there are indeed some that lie dormant "hidden" in our cells, not causing any illness. The mechanism of reactivation occurs when that pathogen emerges from its dormant phase and becomes active again, potentially replicating and spreading, causing illness. There are sporadic reports to date of clinical COVID-19 virus reactivation. Ye *et al.* reported five patients with clinical reactivation presenting mostly with fatigue and fever, but none of them developed severe COVID-19 pneumonia or died [11]. Ravioli *et al.* reported two elderly patients who developed COVID-19, recovered and tested negative by PCR, and then developed a new COVID-19 pneumonia, with one patient dying and the other remaining hospitalized at the time of the report [12]. Lancman *et al*. reported the case of a patient undergoing treatment for CD20-positive B-cell acute lymphoblastic leukemia who experienced a viral reactivation after receiving rituximab and other immunosuppressive chemotherapy [13]. The Korea Disease Control and Prevention Agency has documented 91 cases of recurrent infection, and they emphasize the importance of viral reactivation especially in immunocompromised patients [4].

SARS-CoV-2 reinfection is another possibility in recurrent infection. There are 80 distinct known genotypic variants of this virus; thus reinfection by another variant can lead to COVID-19 reinfection [14]. In a recent case series from the UK, six possible cases of recurrent infection with the possibility of SARS-CoV-2 reinfection were reported, with patients exhibiting long intervals (84 days) between the two COVID-19 episodes. In our patient, both the first and second events were mild and showed no difference in symptom severity; similar clinical cases have been reported from Belgium, the Netherlands, and Hong Kong. These findings are contrary to those reported from Nevada (USA) and Ecuador, where secondary infection was severe [15].

The immunopathogenesis of SARS-CoV-2 involves both cell-mediated and humoral immunity, and the pathogenesis underlying reinfection is still under study. Reactivation is explained by the fact SARS-CoV-2 enters the lung cells via the angiotensin-converting enzyme 2 (ACE2) receptor. ACE2 receptors are expressed in nearly all human organs. In the respiratory system, ACE2 is mainly expressed in type II alveolar epithelial cells, but is weakly expressed on the surface of epithelial cells in the oral and nasal mucosa, indicating that the lower respiratory tract is the primary target of SARS-CoV-2. ACE2 receptors are highly expressed in myocardial cells, kidney, and urinary bladder, and are abundantly expressed in the small intestine, especially in the ileum. The cell-free and macrophage-phagocytosed virus can spread to other organs and infect ACE2-expressing cells at other body sites [16]. Thus, even if COVID-19 patients become SARS-CoV-2-negative by RT-PCR testing, there is still a chance that the patient is harboring active virus that can reactivate at the first possible opportunity. Further, several studies have documented reinfection in COVID-19 patients with new strains of SARS-CoV-2 and have documented distinct infecting strains using next-generation sequencing. Reinfection with variants of concern (including B.1.1.7 and B.1.351) has also been documented following infection with wild-type virus [17, 18].

There is an urgent need for in-depth studies on recurrent COVID-19 infections in order to understand the factors, clinical conditions, and possibility of reinvasion of the virus in the human body. Theoretically, COVID-19 reinfection and reactivation can be identified by whole genome sequencing of the SARS-CoV-2 virus in both the primary and secondary episodes. In reactivation, the strain is the same, whereas in reinfection the strains are different. Recurrent COVID-19 is an emerging problem, and post-discharge and post-recovery surveillance of signs and symptoms in COVID-19-positive patients, with retesting for SARS-CoV-2 in those who present recurrent clinical manifestations of the disease, should be implemented. Further, it is important to stress to these patients the importance of maintaining the use of personal protective equipment and hand hygiene even after being cured of COVID-19.

## Data Availability

Not applicable.
